# Epigenetic Lymphocyte Subset Associations Across Allergic Phenotypes in a Predominantly Pediatric Cohort

**DOI:** 10.3390/diagnostics16203432

**Published:** 2026-10-21

**Authors:** Ergun Mete, Buket Başa Akdoğan, Süleyman Utku Uzun, Cemalettin Dost Zeyrek, Feza Kırbıyık, Ahmet Akçay

**Affiliations:** 1 Department of Medical Microbiology, Faculty of Medicine, Pamukkale University, 20160 Denizli, Türkiye; 2 Immunology and Allergy, Istanbul Allergy Center, 34718 Istanbul, Türkiye; drbuketakdogan@yahoo.com; 3 Department of Public Health, Faculty of Medicine, Pamukkale University, 20160 Denizli, Türkiye; suzun@pau.edu.tr; 4 Pediatric Immunology and Allergy, Istanbul Allergy Center, 34718 Istanbul, Türkiye; dostzeyrek@gmail.com (C.D.Z.); drahmetakcay@gmail.com (A.A.); 5 Clinical Biochemistry, Istanbul Allergy Center, 34718 Istanbul, Türkiye; fkirbiyik@gmail.com

**Keywords:** allergic rhinitis, atopic dermatitis, DNA methylation, epigenetic profiling, food allergy, lymphocyte subsets, pediatric allergy

## Abstract

**Background/Objectives:** DNA methylation-based lymphocyte enumeration may provide an alternative to conventional immunophenotyping, but its associations with allergic phenotypes require careful interpretation. We examined these associations in a predominantly pediatric cohort. **Methods:** This retrospective study included 206 participants aged 1–57 years (189 aged <18 years; 57.8% male; median age 5 years, IQR 3–8), evaluated between 24 November 2025 and 28 February 2026. Absolute subset counts were derived from the epigenetic i.Mune TBNK assay and CBC lymphocyte counts. Group comparisons used Mann–Whitney U tests with Benjamini–Hochberg correction across 56 comparisons. Firth models adjusted for three subset counts, CD4/CD8 ratio, log(IgE+1), age, and sex; percentage-based, comorbidity-adjusted, and age-restricted sensitivity analyses were added. IgE-containing models included 201 complete cases. **Results:** Allergic rhinitis was associated with lower CD4+ counts in the primary model (OR per 100 cells/µL 0.923; *p* = 0.0009), with the association retained after comorbidity adjustment and age restriction but not in the percentage model. Atopic dermatitis was associated with log(IgE+1) (OR 1.475; *p* = 0.0009). Food allergy was associated with higher CD19+ counts in the primary model (OR per 100 cells/µL 1.060; *p* = 0.0341), but this association was not retained in sensitivity analyses. No absolute NK-count difference was found for food allergy. Single-marker discrimination was modest (CD4+ AUC for rhinitis 0.711). **Conclusions:** These exploratory associations are sensitive to the measurement scale and model specification. They do not establish phenotype-specific epigenetic mechanisms, standalone diagnostic utility, or functional immune monitoring.

## 1. Introduction

Asthma, allergic rhinitis, atopic dermatitis, and food allergy are among the most common chronic immune-mediated disorders of childhood, and their burden continues to rise worldwide [1]. They are habitually grouped together under atopy; yet, they differ considerably in clinical course, treatment response, and underlying immunology. Phenotypes that look alike in clinic may arise from distinct molecular endotypes [2], which is why profiling tools able to capture variation between subtypes have drawn attention.

Peripheral lymphocyte profiles may reflect systemic immune regulation in allergic disease, but they cannot be read as simple cell counts. Several mechanisms interact to shape allergic inflammation: Th1/Th2 balance, Th17-mediated inflammation, regulatory T- and B-cell function, memory lymphocyte compartments, and innate lymphoid cell axes [3,4,5]. A shift in one circulating subset may therefore signal redistribution, regulatory imbalance, or trafficking into tissue rather than uniform activation or suppression.

Flow cytometry remains the conventional immunophenotyping method, but it requires fresh samples, standardized processing, and technical expertise. In pediatric practice, these constraints are not trivial [6,7,8]. Quantification-based on DNA methylation offers an alternative. It relies on lineage-specific epigenetic signatures, works on small or frozen samples, and agrees closely with flow cytometry across independent cohorts [7,9]. Age complicates interpretation further: lymphocyte subset counts vary strongly through childhood, and reference studies that include Turkish pediatric data show that age must be taken into account [10,11,12].

We measured peripheral lymphocyte subsets with the epigenetic i.Mune TBNK assay in a predominantly pediatric, mixed-age cohort, examined associations with physician-recorded allergic phenotypes, and estimated apparent single-marker discrimination. The methylation assay was used to enumerate cell lineages rather than to establish disease-specific methylation mechanisms. Percentage-based, comorbidity-adjusted, and age-restricted analyses were undertaken during revision to assess the robustness of the findings.

## 2. Materials and Methods

### 2.1. Study Design and Population

This retrospective study evaluated 206 participants who underwent immunological evaluation between 24 November 2025 and 28 February 2026. Record review was conducted in collaboration with a private outpatient office, the private Barbaros Laboratory, and the Department of Medical Microbiology, Pamukkale University Faculty of Medicine. The cohort comprised 189 participants aged <18 years (91.7%) and 17 adults (8.3%); the age range was 1–57 years. All 206 were retained in the primary analyses. Participants aged <18 years were additionally analyzed as an exploratory sensitivity cohort. The available-record sample had no prospective sample-size calculation; whether records constituted a consecutive series was not documented. The STARD reporting checklist, including explicitly identified reporting limitations, is provided as Supplementary File S2.

Patients with active systemic infection or malignancy were excluded according to the original study description. The supplied analytical cohort contains complete age, sex, phenotype, and lymphocyte data, but five missing total IgE values. These five participants were retained in analyses not requiring IgE and omitted from IgE-containing models; no imputation was performed. All five were aged <18 years (2, 3, 4, 7, and 12 years). None of the 17 adults had missing IgE. The numbers screened before cohort assembly and excluded for each eligibility criterion were not available in the analytical records. Supplementary Figure S1 shows the documented analytical flow.

### 2.2. Clinical Definitions

Clinical phenotypes were extracted from medical records and specialist evaluation, described in the original study records as EAACI-based. Asthma, allergic rhinitis, atopic dermatitis, urticaria, food allergy, wheezy-child phenotype, and primary immunodeficiency were encoded as present or absent. These are physician-recorded diagnostic categories; diagnoses were not independently re-adjudicated using a study-specific reference standard. Asthma and wheezy-child phenotype were retained as separate recorded labels, rather than equating wheeze with established asthma. Primary immunodeficiency subtypes and their diagnostic criteria were not captured in the minimal dataset. Disease severity, treatment exposure, and symptom duration were unavailable for modeling. Diagnoses were not mutually exclusive: 136 participants had at least two of the six allergic/respiratory labels. Individual oral food challenge results, allergen-specific IgE, and skin-prick-test results were not available as structured variables for verification. Consequently, the food-allergy group cannot be described as uniformly challenge-proven or distinguished reliably by diagnostic certainty. Contemporary EAACI guidance places clinical history and sensitization testing within the diagnostic pathway and uses oral food challenge as the reference procedure when needed [13]. We acknowledge the resulting potential for diagnostic misclassification. Sensitivity models adjusted for the five other recorded allergic/respiratory conditions; primary immunodeficiency was not added as a comorbidity covariate.

### 2.3. Laboratory Measurements and Epigenetic Immune Profiling

Unlike conventional flow cytometry, lymphocyte subsets were quantified using the epigenetic Epiontis i.Mune TBNK assay (Epiontis GmbH, Berlin, Germany). This quantitative real-time polymerase chain reaction (PCR) method detects unmethylated DNA regions specific to immune cell lineages following bisulfite conversion. The assay quantified CD3+ T lymphocytes, CD3+CD4+ helper T lymphocytes, CD3+CD8+ cytotoxic T lymphocytes, CD19+ B lymphocytes, and CD16+/CD56+ natural killer (NK) cells. DNA methylation-based immune cell counting has been validated as a reliable and reproducible approach for immunodiagnostic leukocyte quantification, demonstrating high concordance with flow cytometry across multiple independent cohorts [7,8,9].

### 2.4. Calculation of Absolute Counts

Absolute counts for each lymphocyte subset were derived using total lymphocyte counts obtained from complete blood count analysis according to the following formula: absolute subset count (cells/µL) = (epigenetic percentage/100) × absolute lymphocyte count.

### 2.5. Immunoglobulin E Measurement

Total serum immunoglobulin E (IgE) levels were measured using standardized immunoassay techniques. Because IgE values showed a markedly right-skewed distribution, log(IgE+1) transformation was applied before inferential analyses to stabilize variance and reduce the influence of extreme values.

### 2.6. Statistical Analysis

Analyses were independently recalculated during revision using Python 3.12.14, NumPy 2.3.5, SciPy 1.17.0, and pandas 2.2.3; the original analyses had been reported using R 4.3.2. Continuous data are summarized as median (Q1–Q3) and, where relevant, mean ± SD. The eight group-comparison parameters were CD3+, CD4+, CD8+, CD19+, and NK absolute counts, total lymphocytes, CD4/CD8 ratio, and log(IgE+1). Two-sided asymptotic Mann–Whitney U tests, with tie and continuity correction, were used for seven phenotypes. Benjamini–Hochberg correction was explicitly applied across all 56 comparisons as the primary FDR family during revision; raw *p*-values and adjusted q-values are both reported. Within-phenotype correction across eight parameters is additionally provided in Table S1. Cohen’s d was recalculated as the present-minus-absent mean difference divided by the pooled sample SD; it is descriptive and is not the effect-size analogue of the rank test.

Firth penalized logistic regression [14] was used for univariate and multivariable models. Primary multivariable models included CD4+, CD19+, and NK absolute counts, CD4/CD8 ratio, log(IgE+1), age, and sex for asthma, allergic rhinitis, atopic dermatitis, food allergy, and wheezy-child phenotype; these seven covariates were retained regardless of univariate significance. No multivariable model was interpreted for the seven-case primary-immunodeficiency subgroup or the 11-case urticaria subgroup. All IgE-containing primary models used 201 complete cases; age-restricted models used 184 of 189 participants. Odds ratios for absolute counts are expressed per 100 cells/µL; percentage-model ORs are per one percentage point. Model *p*-values use penalized likelihood-ratio tests. Profile penalized-likelihood confidence intervals are reported for significant primary/univariate estimates; complete supplementary coefficient tables also provide explicitly labelled Wald intervals and Wald *p*-values. Regression *p*-values were not multiplicity-adjusted and are exploratory. VIFs were computed from the complete-case predictor matrix. Percentage sensitivity models replaced the three absolute counts with percentages. Comorbidity models added the five other allergic/respiratory labels; multiple deterministic starting values were used to check their penalized-likelihood solutions. These analyses and the age restriction were undertaken during revision; no phenotype clustering was inferred from this small, overlapping cohort.

ROC analyses examined the six absolute-count measures and retained the highest apparent AUC per phenotype. The direction of association, marker, and Youden J-optimal threshold were chosen in the same dataset, so performance estimates are susceptible to optimism. AUC confidence intervals were calculated using the two-sample DeLong variance. Threshold direction and classification counts are reported in Table S6. These analyses describe exploratory discrimination of recorded labels, not validation of the epigenetic assay against conventional immunophenotyping. No independent validation cohort or longitudinal functional monitoring was available.

## 3. Results

### 3.1. Study Population and Baseline Immune Profile

The cohort included 206 participants (119 male, 57.8%), with a median age of 5 years (IQR 3–8) and mean age 8.44 ± 10.68 years. Seventeen participants were adults. The age-restricted cohort included 189 participants aged 1–16 years (109 male, 57.7%; median age 5 years, IQR 3–7). Five participants lacked total IgE; their ages were 2, 3, 4, 7, and 12 years. Table 1 reports parameter-specific sample sizes. Allergic rhinitis was the most frequent recorded phenotype (120/206, 58.3%). Table 2 includes demographic features for each phenotype, while Table S2 provides both present and absent groups, cell-count medians, and overlap counts. The observed demographic differences do not rule out age-related confounding.

### 3.2. Group Comparisons Across Clinical Phenotypes

After BH correction across 56 comparisons, allergic rhinitis remained associated with lower absolute CD3+, CD4+, CD8+, CD19+, and total lymphocyte counts (Table 3). For CD4+, the median was 1899.8 cells/µL in present cases versus 2488.1 in absent cases (raw *p* < 0.001; q < 0.001). The CD19+ comparison had q = 0.0011 and CD8+ had q = 0.0162. These are unadjusted group comparisons, distinct from the multivariable models.

**Figure 1:**
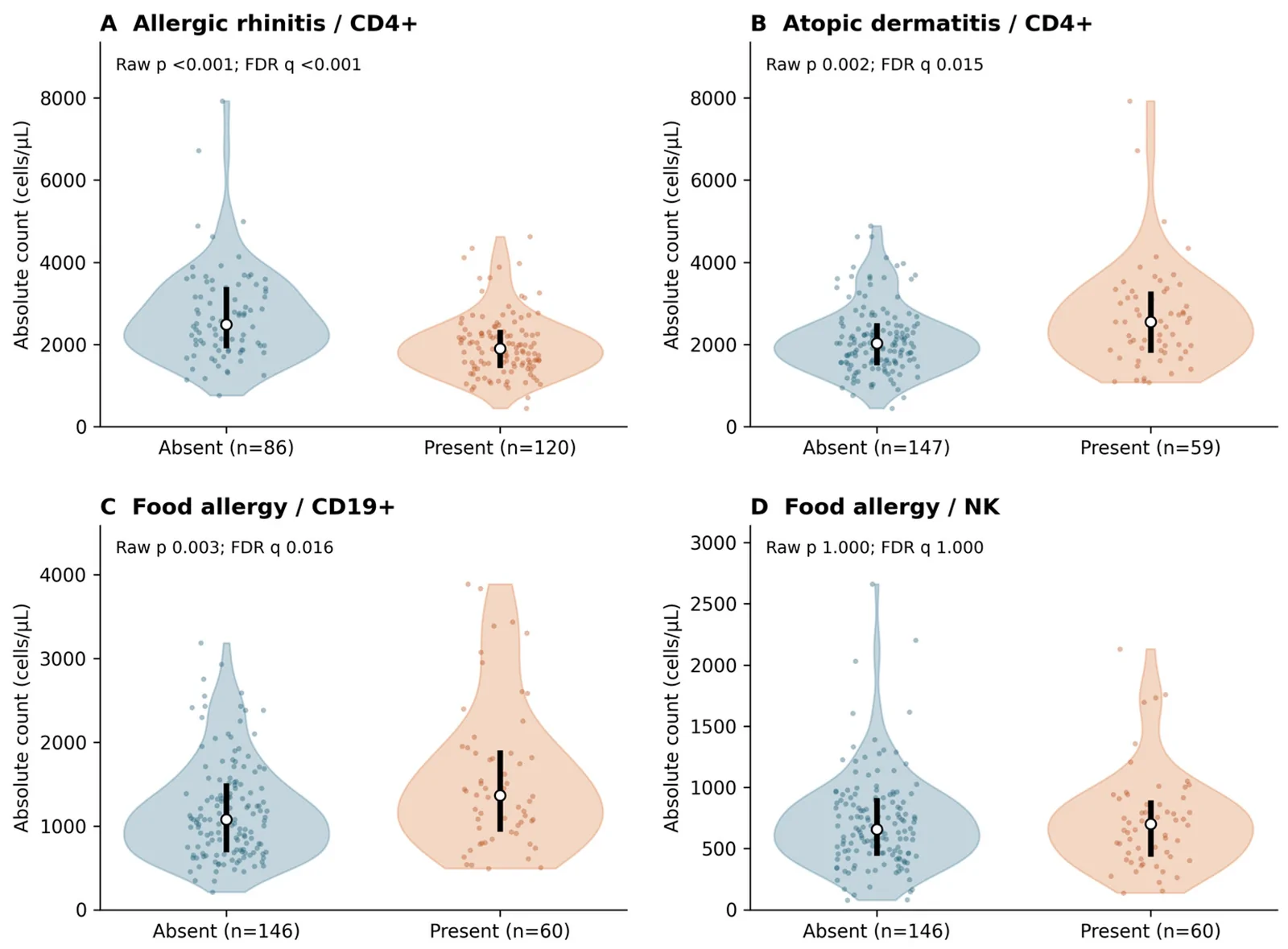
Absolute subset counts by recorded phenotype. Each point represents one participant; violin shapes show kernel-density estimates, white markers show medians and thick lines show Q1–Q3. Panels show rhinitis/CD4+, atopic dermatitis/CD4+, food allergy/CD19+, and food allergy/NK. Raw *p* and global FDR q-values are displayed. NK counts were not significantly different. All plotted measurements come from the study dataset.

Atopic dermatitis was associated with higher CD3+, CD4+, CD19+, and total lymphocyte counts, and higher log(IgE+1), after the global FDR correction. Food allergy was associated with higher CD3+, CD4+, CD19+, and total lymphocyte counts (q = 0.0106–0.0325). Contrary to the original abstract wording, absolute NK counts did not differ by food-allergy status (raw *p* = 1.000; q = 1.000). No asthma subset comparison remained significant after global FDR correction. The seven-case primary-immunodeficiency CD19+ comparison had raw *p* = 0.0499 but q = 0.1393, and was not retained as an FDR-significant finding. Table S1 reports all 56 comparisons, including non-significant results. Figure 1 visualizes the principal cell-count comparisons and the null NK comparison.

### 3.3. Regression Analyses

Selected univariate Firth results are shown in Table 4; all 70 predictor–phenotype estimates, including non-significant results, are provided in Table S4B. These univariate regression *p*-values are exploratory and were not FDR-corrected. Scaling the OR to 100 cells/µL resolves the apparent OR≈1 presentation problem without changing the fitted association or its *p*-value.

Primary seven-covariate Firth models identified lower CD4+ counts with allergic rhinitis (OR per 100 cells/µL 0.923, profile 95% CI 0.876–0.969; *p* = 0.0009), higher log(IgE+1) with atopic dermatitis (OR 1.475, profile 95% CI 1.167–1.904; *p* = 0.0009), higher CD19+ counts with food allergy (OR per 100 cells/µL 1.060, profile 95% CI 1.004–1.120; *p* = 0.0341), and younger age with the wheezy-child phenotype (OR per year 0.538; *p* < 0.001). No TBNK variable was significant in the adjusted asthma model. Table 5 contains selected primary results; all coefficients are in Table S4A. Maximum primary-model VIF was 2.210 (CD4+); shared derivation from total lymphocytes did not, by itself, demonstrate severe multicollinearity. Low VIFs do not establish model stability or exclude confounding.

In percentage sensitivity models, the rhinitis–CD4+ percentage association was not significant (*p* = 0.5621), nor was the food allergy–CD19+ percentage association (*p* = 0.2520); the atopic dermatitis–log(IgE+1) association remained (OR 1.416; *p* = 0.0024). With additional adjustment for the five other allergic/respiratory labels, the rhinitis–CD4+ association remained (OR per 100 cells/µL 0.922; *p* = 0.0081), whereas the food allergy–CD19+ association was not significant (*p* = 0.2218). Thus, the subset associations depended on absolute abundance and, for food allergy, model specification. These models remain vulnerable to sparse events, particularly for the wheezy-child phenotype. The complete coefficients and VIFs are in Tables S5A and S5B.

In the age-restricted models, the rhinitis–CD4+ association remained (OR per 100 cells/µL 0.927; penalized likelihood-ratio *p* = 0.0029), as did atopic dermatitis–log(IgE+1) (OR 1.563; *p* = 0.0004). The food allergy–CD19+ association was not retained (OR per 100 cells/µL 1.032; *p* = 0.2728). Younger age remained associated with the wheezy-child phenotype, and no TBNK predictor was significant for asthma. These *p*-values are penalized likelihood-ratio tests rather than Wald tests; the corresponding Wald values for rhinitis/CD4+ and food allergy/CD19+ were 0.0054 and 0.2851. Table S3 provides both tests to distinguish inferential conventions. Differences in significance do not constitute a formal age-interaction test.

### 3.4. ROC Analysis and Age-Related Immune Maturation

The apparent single-marker AUCs were modest: CD4+ yielded 0.711 for rhinitis (95% CI 0.640–0.783), 0.639 for atopic dermatitis, 0.646 for food allergy, and 0.599 for asthma. For rhinitis, the exploratory threshold <2353.8 cells/µL gave a 77.5% sensitivity and 57.0% specificity. The seven-case primary-immunodeficiency CD19+ AUC was 0.719 (95% CI 0.590–0.847), but this estimate is fragile, selected from multiple markers, and strictly exploratory. It does not support conclusions about immunodeficiency diagnosis. The age-restricted rhinitis/CD4+ AUC was 0.718. Table 6 gives the selected thresholds; Table S6 gives classification counts and direction. None was externally validated.

Age-stratified descriptive medians are shown in Table 7. Counts tended to be higher in the youngest group, but the medians were not uniformly monotonic across all age groups. These are within-cohort summaries, not healthy reference intervals. The ≥18-year group comprises 17 adults and must not be interpreted using pediatric reference values.

Table 8 summarizes the principal primary-model associations and their sensitivity-analysis qualifications.

## 4. Discussion

The methylation-based assay enumerated broad circulating cell lineages in a predominantly pediatric cohort that included 17 adults. It did not measure disease-specific epigenetic mechanisms or lymphocyte function. The most persistent absolute-count association was rhinitis with a lower CD4+, retained after additional diagnosis adjustment and age restriction, but absent when expressed as a CD4+ percentage. Atopic dermatitis tracked with IgE across sensitivity models. Food allergy–CD19+ was an exploratory primary-model association that was not retained with percentages, additional diagnoses, or age restriction. These qualifications prevent a claim of robust phenotype-specific signatures.

The concurrent lower absolute T-cell, B-cell, and total lymphocyte counts in rhinitis may reflect differences in the overall circulating abundance, redistribution, treatment, or other unmeasured factors. Altered circulating T-cell subsets and mucosal lymphocyte profiles have been described in rhinitis [15,16], and peripheral ratios and B-cell subsets differ in some published cohorts [17,18,19]. Those studies provide biological context but cannot establish the mechanism in our cohort. The non-significant percentage-model CD4+ association is particularly relevant: an absolute-count difference need not represent a change in relative cell composition. Neither tissue migration nor systemic immune suppression can be inferred from the current cross-sectional counts.

Food allergy showed higher absolute CD19+ counts in group comparisons and the primary model, but not in the percentage, comorbidity-adjusted, or age-restricted models. Published studies have identified activated B-cell and memory-cell differences in food allergy [20,21,22,23], including cells with potential for pathogenic IgE switching [22]; these finer phenotypes were not measured here. The management literature [24] supports the clinical relevance of an established food allergy but does not validate total CD19+ counts as a diagnostic marker. Our food-allergy result should therefore remain hypothesis-generating and should not be generalized as a robust pediatric B-cell signature.

Atopic dermatitis was associated with higher absolute counts in unadjusted comparisons, while log(IgE+1) remained associated in adjusted and sensitivity models. Pediatric studies describe B-cell subset alterations [25] and cutaneous Th2/Th17 polarization [26]. The current TBNK assay did not resolve those compartments or disease severity, so the IgE association cannot establish a specific peripheral endotype or a mechanism of skin inflammation.

No adjusted TBNK association was found for asthma. Functional T-cell and regulatory B-cell abnormalities reported in asthma and rhinitis [27,28,29,30] may not be captured by broad cell enumeration, but a null count association does not demonstrate normal immune function. Younger age was associated with the wheezy-child label, consistent with the age dependence and heterogeneity of childhood respiratory phenotypes. Primary-immunodeficiency analyses were based on only seven cases; its nominal unadjusted CD19+ comparison did not survive FDR correction, and the AUC result was excluded from the conclusions. Subtype heterogeneity and selection effects cannot be assessed in this subgroup.

Age remains an important interpretive factor [10,11,12]. The cohort spans 1–57 years, and a single linear age covariate does not necessarily capture maturation and later-life changes. Restriction to participants aged <18 years retained the rhinitis–CD4+ and atopic dermatitis–IgE associations but attenuated food allergy–CD19+. Additional age associations emerged within the restricted models. These findings support cautious interpretation rather than proving that age confounding has been eliminated.

Epigenetic enumeration may offer sample-handling advantages over conventional immunophenotyping [6,7,8,9], but this study did not conduct a head-to-head assay validation, assess the cost or turnaround time, or follow changes during treatment. As with other allergy biomarker studies [30,31], clinical utility requires a prospective evaluation within a multiparameter framework. Apparent AUCs around 0.6–0.7 and data-selected thresholds are insufficient for a standalone diagnosis.

Limitations include retrospective selection from available records, no healthy controls, no external validation, unmeasured severity and treatment, and uncertain diagnostic adjudication. Food-allergy labels could not be verified as uniformly oral-challenge-proven; primary-immunodeficiency subtypes were unavailable. The 24 November–28 February window lasted approximately three months and may introduce winter-season bias; it cannot capture chronic disease trajectories. Participants had overlapping conditions, and adding binary comorbidity indicators does not replace an adequately powered phenotype-cluster analysis. Several models have sparse events relative to the number of predictors; Firth penalization and low VIFs do not remove overfitting or guarantee stability. Missing IgE was handled by a complete-case analysis without assuming missingness at random. The 17 adults limit exclusively pediatric inference. Sensitivity analyses and the explicit 56-test FDR family were specified during revision rather than prospectively. The ROC marker and threshold selection used the same data, creating potential optimism. Finally, lineage enumeration does not measure cytokine production, activation, antigen-specific responses, or tissue inflammation; functional or longitudinal immune-monitoring claims are unsupported.

## 5. Conclusions

Epigenetic enumeration showed exploratory associations with recorded allergic phenotypes in a predominantly pediatric, mixed-age cohort. The rhinitis–absolute CD4+ association persisted after additional diagnosis adjustment and age restriction, but not in the percentage model. Atopic dermatitis was associated with IgE across sensitivity models. Food allergy–CD19+ was not robust to these alternative analyses. Findings in the seven-case primary-immunodeficiency subgroup do not support the conclusions. Prospective, diagnostically well-characterized cohorts with functional measurements and independent validation are needed before diagnostic or immune-monitoring applications can be inferred.

## Figures and Tables

**Table 1:** Participant characteristics and lymphocyte subset summary (*n* = 206).

Parameter	*n*	Mean ± SD	Median	Q1–Q3	Min–Max	Unit
Age	206	8.44 ± 10.68	5.00	3.00–8.00	1.00–57.00	years
NK	206	717.73 ± 386.02	667.73	462.83–889.76	78.57–2660.74	cells/µL
CD19+	206	1295.53 ± 693.99	1114.32	787.39–1573.47	211.93–3887.94	cells/µL
CD4+	206	2281.79 ± 994.69	2102.39	1611.68–2716.54	446.80–7927.95	cells/µL
CD3+	206	3256.99 ± 1295.84	2998.92	2402.58–3942.41	648.99–10,168.43	cells/µL
CD4/CD8	206	2.13 ± 0.99	1.90	1.54–2.45	0.44–7.38	–
CD8+	206	1189.80 ± 557.30	1102.26	848.72–1425.74	269.79–4498.45	cells/µL
Total lymphocytes	206	5338.62 ± 1974.56	5008.00	3924.75–6436.00	1178.00–14,877.00	cells/µL
log(IgE+1)	201	4.81 ± 1.49	4.94	3.97–5.70	1.01–8.04	–
Total IgE	201	318.70 ± 512.41	139.00	52.00–298.00	1.75–3100.00	IU/mL

SD, standard deviation; Q1–Q3, 25th–75th percentiles; NK, natural killer cells. Total IgE and log(IgE+1): *n* = 201; all other parameters: *n* = 206. Percentiles use linear interpolation.

**Table 2:** Recorded diagnoses and demographic characteristics of participants with each condition.

Condition	Present *n*	Absent *n*	Cohort %	Male *n* (%)	Age Median (IQR)	Adults *n*	Missing IgE *n*
Asthma	94	112	45.6	52 (55.3)	6.0 (5.0–9.0)	7	2
Allergic rhinitis	120	86	58.3	69 (57.5)	6.0 (5.0–9.0)	9	3
Atopic dermatitis	59	147	28.6	34 (57.6)	4.0 (2.0–6.0)	2	3
Urticaria	11	195	5.3	6 (54.5)	7.0 (3.5–13.0)	2	0
Food allergy	60	146	29.1	35 (58.3)	4.0 (2.0–7.0)	2	3
Wheezy-child phenotype	16	190	7.8	11 (68.8)	2.0 (1.0–4.0)	0	0
Primary immunodeficiency	7	199	3.4	6 (85.7)	3.0 (2.0–4.0)	0	0

Conditions overlap; counts must not be summed as mutually exclusive groups. Male percentages and age summaries refer to present cases. Adult denotes age ≥18 years. IQR, interquartile range.

**Table 3:** Group differences retained after Benjamini–Hochberg correction across 56 comparisons.

Phenotype/Marker	Absent Median (IQR)	Present Median (IQR)	Raw *p*	FDR q	d
Allergic rhinitis/CD3+	3632.9 (2768.4–4370.3)	2668.5 (2211.5–3531.8)	<0.001	<0.001	−0.656
Allergic rhinitis/CD4+	2488.1 (1974.8–3330.6)	1899.8 (1499.7–2290.5)	<0.001	<0.001	−0.750
Allergic rhinitis/CD8+	1222.6 (915.3–1652.6)	1028.4 (759.9–1281.4)	0.0029	0.0162	−0.392
Allergic rhinitis/CD19+	1341.9 (992.6–1850.1)	1028.5 (686.1–1371.4)	<0.001	0.0011	−0.529
Allergic rhinitis/Total lymphocytes	5665.0 (4567.2–6914.0)	4585.5 (3651.8–5707.8)	<0.001	<0.001	−0.597
Atopic dermatitis/CD3+	2831.6 (2374.5–3671.1)	3733.0 (2683.8–4271.5)	0.0032	0.0162	0.470
Atopic dermatitis/CD4+	2031.9 (1569.4–2458.4)	2549.8 (1866.6–3229.1)	0.0019	0.0150	0.551
Atopic dermatitis/CD19+	1079.9 (720.2–1451.8)	1296.9 (945.3–1903.2)	0.0103	0.0410	0.411
Atopic dermatitis/Total lymphocytes	4806.0 (3811.0–6054.5)	5579.0 (4521.0–6928.0)	0.0032	0.0162	0.490
Atopic dermatitis/log(IgE+1)	4.8 (3.9–5.5)	5.2 (4.3–6.2)	0.0112	0.0419	0.443
Food allergy/CD3+	2831.3 (2350.1–3726.6)	3632.9 (2753.3–4375.6)	0.0011	0.0106	0.562
Food allergy/CD4+	2032.0 (1546.8–2518.5)	2410.8 (1866.3–3347.9)	0.0010	0.0106	0.581
Food allergy/CD19+	1077.0 (718.7–1478.6)	1362.8 (965.0–1870.1)	0.0030	0.0162	0.552
Food allergy/Total lymphocytes	4892.5 (3727.2–5999.5)	5500.5 (4325.5–7015.0)	0.0076	0.0325	0.503
Wheezy-child phenotype/log(IgE+1)	5.0 (4.0–5.8)	4.3 (3.0–4.5)	0.0046	0.0216	−0.711

Values are medians (Q1–Q3). Absolute counts are cells/µL; log(IgE+1) is unitless. Raw *p*-values are two-sided asymptotic Mann–Whitney tests; q-values are BH-adjusted across all 56 tests. Cohen’s d uses the pooled sample SD and a present-minus-absent sign. All results, including non-significant comparisons, appear in Table S1.

**Table 4:** Univariate Firth penalized logistic regression (selected results).

Phenotype	Predictor	*n*	OR (Profile 95% CI)	PLR *p*
Asthma	CD3+	206	0.974 (0.951–0.996)	0.0220
Asthma	CD4+	206	0.958 (0.926–0.988)	0.0052
Asthma	CD19+	206	0.957 (0.916–0.997)	0.0332
Asthma	Total lymphocytes	206	0.985 (0.970–0.999)	0.0412
Asthma	CD4/CD8	206	0.725 (0.519–0.972)	0.0311
Allergic rhinitis	CD3+	206	0.946 (0.920–0.971)	<0.001
Allergic rhinitis	CD4+	206	0.917 (0.882–0.950)	<0.001
Allergic rhinitis	CD8+	206	0.931 (0.878–0.981)	0.0065
Allergic rhinitis	CD19+	206	0.927 (0.885–0.966)	<0.001
Allergic rhinitis	Total lymphocytes	206	0.969 (0.953–0.984)	<0.001
Atopic dermatitis	CD3+	206	1.035 (1.011–1.061)	0.0034
Atopic dermatitis	CD4+	206	1.053 (1.022–1.090)	<0.001
Atopic dermatitis	CD19+	206	1.056 (1.013–1.103)	0.0099
Atopic dermatitis	Total lymphocytes	206	1.024 (1.008–1.040)	0.0023
Atopic dermatitis	log(IgE+1)	201	1.359 (1.093–1.713)	0.0053
Atopic dermatitis	Age	206	0.946 (0.884–0.988)	0.0082
Food allergy	CD3+	206	1.042 (1.018–1.070)	<0.001
Food allergy	CD4+	206	1.057 (1.025–1.094)	<0.001
Food allergy	CD19+	206	1.076 (1.031–1.125)	<0.001
Food allergy	Total lymphocytes	206	1.024 (1.009–1.041)	0.0016
Food allergy	CD4/CD8	206	1.390 (1.045–1.886)	0.0236
Food allergy	Age	206	0.959 (0.907–0.995)	0.0240
Wheezy-child phenotype	log(IgE+1)	201	0.635 (0.447–0.889)	0.0080
Wheezy-child phenotype	Age	206	0.557 (0.380–0.751)	<0.001

Selected nominally significant results; no regression multiplicity correction was applied. ORs for absolute counts are per 100 cells/µL; age is per year and other continuous variables per one unit. *n* = 201 for log(IgE+1) and 206 otherwise. *p*-values are penalized likelihood-ratio tests; CIs are profile penalized-likelihood intervals. All 70 univariate estimates are in Table S4B.

**Table 5:** Multivariable Firth penalized logistic regression (selected results).

Phenotype	Predictor	OR (Profile 95% CI)	PLR *p*
Allergic rhinitis	CD4+	0.923 (0.876–0.969)	<0.001
Atopic dermatitis	log(IgE+1)	1.475 (1.167–1.904)	<0.001
Food allergy	CD19+	1.060 (1.004–1.120)	0.0341
Wheezy-child phenotype	Age	0.538 (0.342–0.762)	<0.001

Each primary model includes CD4+, CD19+, NK, CD4/CD8 ratio, log(IgE+1), age, and sex; *n* = 201 for every model. Cell-count ORs are per 100 cells/µL. Selected nominally significant results are shown; *p*-values are penalized likelihood-ratio tests and CIs are profile intervals. Complete coefficients, including non-significant results and event counts, are in Table S4A.

**Table 6:** Exploratory apparent discrimination of recorded phenotypes by selected single markers.

Phenotype/Marker	AUC (95% CI)	Threshold	Sens. %	Spec. %	J
Asthma/CD4+	0.599 (0.522–0.676)	<2787.6	88.3	30.4	0.187
Allergic rhinitis/CD4+	0.711 (0.640–0.783)	<2353.8	77.5	57.0	0.345
Atopic dermatitis/CD4+	0.639 (0.552–0.725)	>2539.8	52.5	78.2	0.308
Urticaria/CD19+	0.614 (0.430–0.798)	<859.6	54.5	71.3	0.258
Food allergy/CD4+	0.646 (0.562–0.729)	>2787.6	38.3	84.9	0.233
Wheezy-child phenotype/Total lymphocytes	0.646 (0.506–0.786)	>6371.0	56.2	76.3	0.326
Primary immunodeficiency/CD19+	0.719 (0.590–0.847)	>1292.1	85.7	60.8	0.465

The highest apparent AUC among six absolute-count measures is shown per phenotype. Marker, direction, and Youden J threshold were selected in the same dataset; estimates are unvalidated and may be optimistic. Cutoffs are cells/µL. CIs use DeLong variance. Primary immunodeficiency (*n* = 7) and urticaria (*n* = 11) are especially uncertain.

**Table 7:** Age-stratified median lymphocyte subset counts in the study cohort (cells/µL).

Age (Years)	*n*	CD3+	CD4+	CD8+	CD19+	NK	CD4/CD8
0–2	35	4109	3097	1520	1856	861	2.08
3–5	71	3106	2046	1061	1224	611	1.98
6–11	68	2789	1803	1052	1037	674	1.78
12–17	15	2517	2000	984	786	463	1.88
≥18	17	2698	1977	990	679	636	1.88

Values are medians in cells/µL. *n*, number of participants; CD, cluster of differentiation; NK, natural killer cells.

**Table 8:** Primary-model associations and sensitivity-analysis qualifications.

Phenotype	Primary Association	Sensitivity Qualification
Allergic rhinitis	Lower absolute CD4+	Retained with comorbidities and age restriction; not with percentages
Atopic dermatitis	Higher log(IgE+1)	Retained across the three sensitivity specifications
Food allergy	Higher absolute CD19+	Not retained with percentages, comorbidities or age restriction
Asthma	No adjusted TBNK association	No TBNK signal after age restriction
Wheezy-child phenotype	Younger age	Sparse events; exploratory

Primary associations refer to the seven-covariate absolute-count models, not to causal effects or validated phenotype signatures. Sensitivity *p*-values are penalized likelihood-ratio tests; regression *p*-values are exploratory and not multiplicity-adjusted.

## Data Availability

The minimal dataset supporting the findings of this study is provided in Supplementary File S1. Additional de-identified data may be available from the corresponding author upon reasonable request, subject to ethical and privacy restrictions protecting participant confidentiality.

## Supplementary Materials

The following supporting information can be downloaded at: https://www.mdpi.com/article/10.3390/diagnostics16203432/s1, Figure S1: Documented analytical flow; Table S1: All group comparisons; Table S2: Demographics and overlapping diagnoses; Table S3: Age-restricted models compared with primary models; Table S4A: Complete primary multivariable results; Table S4B: Complete univariate results; Table S5A: Percentage sensitivity models; Table S5B: Comorbidity-adjusted sensitivity models; Table S6: Exploratory threshold classification counts; File S1: Minimal dataset; File S2: STARD Checklist.
