# Supplementary material for: Epigenetic Lymphocyte Subset Associations Across Allergic Phenotypes in a Predominantly Pediatric Cohort
Source: Diagnostics (Basel). 2026 Oct 21;16(20):3432. doi: 10.3390/diagnostics16203432 (PMC13703722; doi:10.3390/diagnostics16203432)
Supplement: Supplementary file 1 [file diagnostics-16-03432-s001.zip › Supplementary_Tables_S1-S6.pdf]

Supplementary statistical tables and analytical flow

Manuscript ID: diagnostics-4602566
Epigenetic Lymphocyte Subset Associations Across Allergic Phenotypes in a Predominantly Pediatric Cohort

Patient values are unchanged. All recalculations use the supplied minimal dataset. PLR denotes the Firth penalized likelihood-ratio test. Wald p-values are shown separately and must not be substituted for PLR values without changing the stated method. Cell-count ORs are per 100 cells/µL. These regression analyses are exploratory and not multiplicity-adjusted.

Figure S1. Documented analytical flow

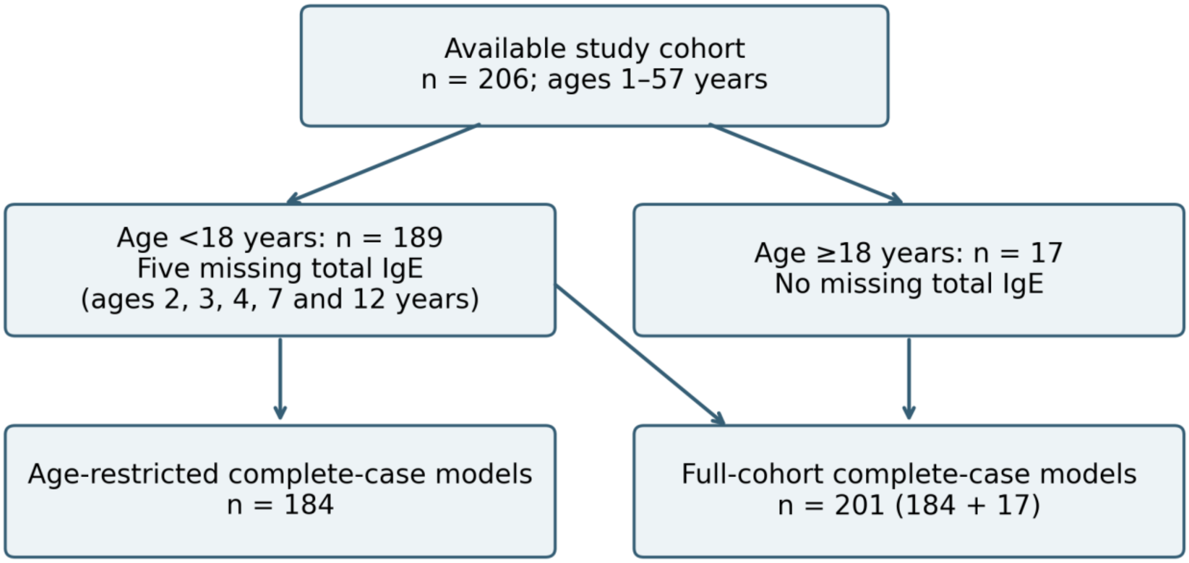

This figure starts with the available analytical cohort; the number screened before assembly and counts excluded for individual eligibility criteria were not recorded in the supplied analysis files. All five missing-IgE records are in the <18-year cohort. The 17 adults have complete IgE.

Table S1. All group comparisons

Primary BH family: 56 tests (seven phenotypes × eight parameters). Within-phenotype q-values are a supplementary eight-test correction. Values are medians (Q1–Q3); n varies only for IgE. Tests use the asymptotic Mann–Whitney procedure with tie and continuity correction. d is pooled-SD Cohen's d, present minus absent.

Asthma

Marker	n present / absent	Absent median (IQR)	Present median (IQR)	Raw p	q 56	q 8	d
CD3+	94/112	3190.8 (2504.3–4162.4)	2821.1 (2356.2–3606.4)	0.0191	0.0630	0.0765	-0.323

Marker	n present / absent	Absent median (IQR)	Present median (IQR)	Raw p	q 56	q 8	d
CD4+	94/112	2220.6 (1671.3–3033.8)	1987.2 (1567.4–2501.0)	0.0144	0.0505	0.0765	-0.391
CD8+	94/112	1119.8 (827.0–1475.3)	1061.1 (881.0–1401.2)	0.9560	0.9867	0.9560	0.078
CD19+	94/112	1193.5 (793.5–1790.3)	1077.3 (741.4–1413.4)	0.0848	0.1845	0.1370	-0.301
NK	94/112	647.7 (476.5–894.3)	710.4 (433.8–880.8)	0.7576	0.8486	0.8659	-0.106
Total lymphocytes	94/112	5182.0 (3973.8–6801.5)	4831.0 (3809.0–5988.2)	0.0856	0.1845	0.1370	-0.288
CD4/CD8	94/112	2.0 (1.6–2.6)	1.9 (1.5–2.2)	0.0510	0.1393	0.1361	-0.304
log(IgE+1)	92/109	4.7 (4.0–5.5)	5.2 (4.0–5.9)	0.1452	0.2593	0.1935	0.160

Allergic rhinitis

Marker	n present / absent	Absent median (IQR)	Present median (IQR)	Raw p	q 56	q 8	d
CD3+	120/86	3632.9 (2768.4–4370.3)	2668.5 (2211.5–3531.8)	<0.001	<0.001	<0.001	-0.656
CD4+	120/86	2488.1 (1974.8–3330.6)	1899.8 (1499.7–2290.5)	<0.001	<0.001	<0.001	-0.750
CD8+	120/86	1222.6 (915.3–1652.6)	1028.4 (759.9–1281.4)	0.0029	0.0162	0.0046	-0.392
CD19+	120/86	1341.9 (992.6–1850.1)	1028.5 (686.1–1371.4)	<0.001	0.0011	<0.001	-0.529
NK	120/86	731.4 (519.6–936.8)	627.5 (423.3–832.2)	0.0400	0.1245	0.0533	-0.277
Total lymphocytes	120/86	5665.0 (4567.2–6914.0)	4585.5 (3651.8–5707.8)	<0.001	<0.001	<0.001	-0.597
CD4/CD8	120/86	2.0 (1.6–2.6)	1.9 (1.5–2.3)	0.1376	0.2593	0.1528	-0.247
log(IgE+1)	117/84	4.6 (3.9–5.3)	5.2 (4.1–5.8)	0.1528	0.2593	0.1528	0.142

Atopic dermatitis

Marker	n present / absent	Absent median (IQR)	Present median (IQR)	Raw p	q 56	q 8	d
CD3+	59/147	2831.6 (2374.5–3671.1)	3733.0 (2683.8–4271.5)	0.0032	0.0162	0.0085	0.470
CD4+	59/147	2031.9 (1569.4–2458.4)	2549.8 (1866.6–3229.1)	0.0019	0.0150	0.0085	0.551
CD8+	59/147	1046.9 (832.8–1394.3)	1176.8 (903.1–1505.9)	0.0796	0.1845	0.1062	0.183
CD19+	59/147	1079.9 (720.2–1451.8)	1296.9 (945.3–1903.2)	0.0103	0.0410	0.0179	0.411
NK	59/147	652.5 (459.8–867.7)	686.3 (469.4–951.1)	0.3023	0.4575	0.3023	0.260
Total lymphocytes	59/147	4806.0 (3811.0–6054.5)	5579.0 (4521.0–6928.0)	0.0032	0.0162	0.0085	0.490
CD4/CD8	59/147	1.9 (1.5–2.4)	2.0 (1.5–2.6)	0.2597	0.4039	0.2967	0.178
log(IgE+1)	56/145	4.8 (3.9–5.5)	5.2 (4.3–6.2)	0.0112	0.0419	0.0179	0.443

Urticaria

Marker	n present / absent	Absent median (IQR)	Present median (IQR)	Raw p	q 56	q 8	d
CD3+	11/195	3012.8 (2412.5–3934.1)	2870.7 (2319.4–3894.2)	0.7315	0.8486	0.7315	-0.031
CD4+	11/195	2103.3 (1613.5–2719.0)	2045.9 (1562.8–2332.9)	0.6661	0.7937	0.7315	-0.151

Marker	n present / absent	Absent median (IQR)	Present median (IQR)	Raw p	q 56	q 8	d
CD8+	11/195	1105.8 (852.8–1427.2)	941.3 (756.4–1289.8)	0.4604	0.5996	0.7315	-0.247
CD19+	11/195	1124.2 (793.3–1596.0)	857.0 (657.8–1328.3)	0.2065	0.3401	0.7315	-0.350
NK	11/195	665.3 (462.9–895.9)	705.5 (465.4–815.2)	0.6068	0.7551	0.7315	-0.250
Total lymphocytes	11/195	5022.0 (3950.0–6430.5)	4696.0 (3439.0–5895.5)	0.3602	0.5172	0.7315	-0.232
CD4/CD8	11/195	1.9 (1.5–2.4)	2.2 (1.5–2.5)	0.6548	0.7937	0.7315	0.128
log(IgE+1)	11/190	4.8 (4.0–5.7)	5.2 (5.0–6.0)	0.0989	0.2051	0.7315	0.551

Food allergy

Marker	n present / absent	Absent median (IQR)	Present median (IQR)	Raw p	q 56	q 8	d
CD3+	60/146	2831.3 (2350.1–3726.6)	3632.9 (2753.3–4375.6)	0.0011	0.0106	0.0045	0.562
CD4+	60/146	2032.0 (1546.8–2518.5)	2410.8 (1866.3–3347.9)	0.0010	0.0106	0.0045	0.581
CD8+	60/146	1053.9 (829.9–1401.2)	1209.0 (887.3–1533.2)	0.1187	0.2374	0.1583	0.206
CD19+	60/146	1077.0 (718.7–1478.6)	1362.8 (965.0–1870.1)	0.0030	0.0162	0.0081	0.552
NK	60/146	658.9 (462.8–889.8)	700.3 (458.4–873.0)	1.0000	1.0000	1.0000	0.031
Total lymphocytes	60/146	4892.5 (3727.2–5999.5)	5500.5 (4325.5–7015.0)	0.0076	0.0325	0.0151	0.503
CD4/CD8	60/146	1.9 (1.5–2.3)	2.0 (1.7–2.7)	0.0841	0.1845	0.1345	0.360
log(IgE+1)	57/144	4.9 (4.0–5.6)	5.0 (4.0–6.0)	0.8118	0.8742	0.9278	0.036

Wheezy-child phenotype

Marker	n present / absent	Absent median (IQR)	Present median (IQR)	Raw p	q 56	q 8	d
CD3+	16/190	2931.1 (2382.9–3837.9)	3797.7 (2685.8–4902.2)	0.0710	0.1806	0.1892	0.414
CD4+	16/190	2055.6 (1607.3–2673.5)	2583.9 (1762.6–3082.8)	0.1502	0.2593	0.3004	0.466
CD8+	16/190	1071.7 (848.7–1425.7)	1131.5 (859.4–1456.3)	0.7482	0.8486	0.7482	0.003
CD19+	16/190	1106.1 (783.8–1538.6)	1306.6 (792.1–1728.8)	0.4539	0.5996	0.5188	0.196
NK	16/190	659.6 (460.6–871.9)	752.7 (555.2–917.6)	0.3908	0.5338	0.5188	0.105
Total lymphocytes	16/190	4927.0 (3898.5–6165.0)	6402.5 (4544.0–7271.2)	0.0523	0.1393	0.1892	0.418
CD4/CD8	16/190	1.9 (1.5–2.4)	2.0 (1.7–2.6)	0.2463	0.3941	0.3941	0.342
log(IgE+1)	16/185	5.0 (4.0–5.8)	4.3 (3.0–4.5)	0.0046	0.0216	0.0371	-0.711

Primary immunodeficiency

Marker	n present / absent	Absent median (IQR)	Present median (IQR)	Raw p	q 56	q 8	d
CD3+	7/199	2985.0 (2391.7–3934.1)	3012.9 (2838.5–3547.6)	0.8365	0.8838	0.9559	-0.061
CD4+	7/199	2065.2 (1608.1–2716.5)	2365.8 (2054.0–3087.8)	0.3496	0.5152	0.7797	0.227
CD8+	7/199	1105.8 (852.8–1427.2)	1039.1 (777.1–1252.6)	0.5400	0.6872	0.8640	-0.260

Marker	n present / absent	Absent median (IQR)	Present median (IQR)	Raw p	q 56	q 8	d
CD19+	7/199	1106.0 (782.9–1562.9)	1530.8 (1406.4–1734.7)	0.0499	0.1393	0.3989	0.477
NK	7/199	670.2 (461.3–890.0)	589.1 (523.8–819.6)	0.9691	0.9867	0.9691	0.070
Total lymphocytes	7/199	5022.0 (3907.0–6426.0)	4957.0 (4716.0–5877.5)	0.7964	0.8742	0.9559	0.105
CD4/CD8	7/199	1.9 (1.5–2.4)	2.1 (1.9–2.9)	0.1503	0.2593	0.6011	0.404
log(IgE+1)	7/194	5.0 (4.0–5.8)	4.6 (3.9–5.0)	0.3898	0.5338	0.7797	-0.291

Table S2. Demographics and overlapping diagnoses

Both present and absent groups are reported. Diagnoses overlap and these summaries do not exclude demographic confounding.

Phenotype / status	n	Male n (%)	Age median (IQR)	Adults n	Missing IgE n
Asthma / present	94	52 (55.3)	6.0 (5.0–9.0)	7	2
Asthma / absent	112	67 (59.8)	4.0 (2.0–7.2)	10	3
Allergic rhinitis / present	120	69 (57.5)	6.0 (5.0–9.0)	9	3
Allergic rhinitis / absent	86	50 (58.1)	3.0 (2.0–6.0)	8	2
Atopic dermatitis / present	59	34 (57.6)	4.0 (2.0–6.0)	2	3
Atopic dermatitis / absent	147	85 (57.8)	6.0 (4.0–9.0)	15	2
Urticaria / present	11	6 (54.5)	7.0 (3.5–13.0)	2	0
Urticaria / absent	195	113 (57.9)	5.0 (3.0–8.0)	15	5
Food allergy / present	60	35 (58.3)	4.0 (2.0–7.0)	2	3
Food allergy / absent	146	84 (57.5)	6.0 (4.0–9.8)	15	2
Wheezy-child phenotype / present	16	11 (68.8)	2.0 (1.0–4.0)	0	0
Wheezy-child phenotype / absent	190	108 (56.8)	6.0 (4.0–9.0)	17	5
Primary immunodeficiency / present	7	6 (85.7)	3.0 (2.0–4.0)	0	0
Primary immunodeficiency / absent	199	113 (56.8)	6.0 (3.0–8.5)	17	5

Median absolute subset counts (cells/µL), present cases:

Phenotype	CD3+	CD4+	CD8+	CD19+	NK	Total lymphocytes
Asthma	2821.1	1987.2	1061.1	1077.3	710.4	4831.0
Allergic rhinitis	2668.5	1899.8	1028.4	1028.5	627.5	4585.5
Atopic dermatitis	3733.0	2549.8	1176.8	1296.9	686.3	5579.0
Urticaria	2870.7	2045.9	941.3	857.0	705.5	4696.0
Food allergy	3632.9	2410.8	1209.0	1362.8	700.3	5500.5
Wheezy-child phenotype	3797.7	2583.9	1131.5	1306.6	752.7	6402.5

Phenotype	CD3+	CD4+	CD8+	CD19+	NK	Total lymphocytes
Primary immunodeficiency	3012.9	2365.8	1039.1	1530.8	589.1	4957.0

Number with both recorded diagnoses; diagonal entries are phenotype totals. At least two of these labels were present in 136/206 participants (66.0%).

Diagnosis	Asthma	Rhinitis	Dermatitis	Urticaria	Food allergy	Wheezy
Asthma	94	78	11	1	10	0
Allergic rhinitis	78	120	16	4	18	3
Atopic dermatitis	11	16	59	1	33	2
Urticaria	1	4	1	11	2	0
Food allergy	10	18	33	2	60	1
Wheezy-child phenotype	0	3	2	0	1	16

Table S3. Age-restricted models compared with primary models

Full cohort: 201 complete cases; age <18: 184. The same seven covariates are used. All CIs in this comparison are Wald intervals based on the inverse expected information at the Firth estimate; the selected primary estimates in the manuscript use profile intervals. Both PLR and Wald tests are provided.

Asthma

Predictor	Full OR (Wald CI)	PLR p	Wald p	<18 OR (Wald CI)	PLR p	Wald p
CD4+	0.979 (0.935–1.025)	0.3467	0.3581	0.980 (0.934–1.028)	0.3962	0.4074
CD19+	0.969 (0.918–1.022)	0.2283	0.2447	0.992 (0.937–1.050)	0.7788	0.7840
NK	1.008 (0.921–1.104)	0.8520	0.8548	1.018 (0.928–1.116)	0.7024	0.7098
CD4/CD8	0.811 (0.567–1.159)	0.2285	0.2504	0.876 (0.599–1.280)	0.4746	0.4926
log(IgE+1)	1.100 (0.903–1.340)	0.3316	0.3439	1.015 (0.817–1.260)	0.8943	0.8965
Age	0.993 (0.967–1.021)	0.6233	0.6391	1.167 (1.048–1.299)	0.0027	0.0049
Male sex	0.774 (0.432–1.386)	0.3803	0.3886	0.783 (0.418–1.466)	0.4363	0.4440

Allergic rhinitis

Predictor	Full OR (Wald CI)	PLR p	Wald p	<18 OR (Wald CI)	PLR p	Wald p
CD4+	0.923 (0.878–0.971)	<0.001	0.0019	0.927 (0.878–0.978)	0.0029	0.0054
CD19+	0.971 (0.919–1.026)	0.2826	0.2943	1.011 (0.952–1.074)	0.7236	0.7280
NK	1.032 (0.940–1.134)	0.4998	0.5061	1.035 (0.938–1.141)	0.4817	0.4948
CD4/CD8	1.077 (0.747–1.553)	0.6810	0.6909	1.248 (0.841–1.853)	0.2484	0.2713
log(IgE+1)	1.082 (0.880–1.331)	0.4464	0.4559	0.953 (0.746–1.216)	0.6912	0.6969
Age	0.995 (0.967–1.024)	0.7181	0.7271	1.310 (1.141–1.503)	<0.001	<0.001
Male sex	0.881 (0.476–1.629)	0.6811	0.6858	0.941 (0.476–1.860)	0.8600	0.8618

Atopic dermatitis

Predictor	Full OR (Wald CI)	PLR p	Wald p	<18 OR (Wald CI)	PLR p	Wald p
CD4+	1.044 (0.995–1.095)	0.0707	0.0825	1.043 (0.993–1.096)	0.0804	0.0935
CD19+	1.023 (0.965–1.083)	0.4407	0.4493	1.010 (0.952–1.072)	0.7328	0.7389
NK	1.014 (0.922–1.114)	0.7722	0.7782	1.012 (0.918–1.115)	0.8130	0.8173
CD4/CD8	0.938 (0.638–1.379)	0.7351	0.7448	0.914 (0.615–1.360)	0.6442	0.6581
log(IgE+1)	1.475 (1.155–1.885)	<0.001	0.0019	1.563 (1.192–2.049)	<0.001	0.0012
Age	0.956 (0.903–1.013)	0.0695	0.1250	0.862 (0.754–0.985)	0.0196	0.0289
Male sex	0.894 (0.457–1.749)	0.7423	0.7445	0.904 (0.451–1.814)	0.7744	0.7772

Food allergy

Predictor	Full OR (Wald CI)	PLR p	Wald p	<18 OR (Wald CI)	PLR p	Wald p
CD4+	1.034 (0.987–1.084)	0.1432	0.1595	1.036 (0.987–1.088)	0.1366	0.1530
CD19+	1.060 (1.003–1.121)	0.0341	0.0394	1.032 (0.974–1.093)	0.2728	0.2851
NK	0.938 (0.852–1.033)	0.1697	0.1919	0.940 (0.851–1.037)	0.1946	0.2182
CD4/CD8	1.138 (0.788–1.644)	0.4787	0.4904	1.107 (0.755–1.624)	0.5906	0.6032
log(IgE+1)	1.064 (0.854–1.325)	0.5712	0.5789	1.189 (0.931–1.518)	0.1519	0.1665
Age	0.983 (0.945–1.023)	0.3720	0.4106	0.862 (0.755–0.983)	0.0176	0.0268
Male sex	1.077 (0.557–2.085)	0.8236	0.8255	0.926 (0.466–1.840)	0.8250	0.8272

Wheezy-child phenotype

Predictor	Full OR (Wald CI)	PLR p	Wald p	<18 OR (Wald CI)	PLR p	Wald p
CD4+	0.991 (0.922–1.065)	0.8058	0.8060	0.991 (0.922–1.065)	0.8058	0.8060
CD19+	0.942 (0.861–1.030)	0.1766	0.1923	0.942 (0.861–1.030)	0.1766	0.1923
NK	1.010 (0.883–1.157)	0.8796	0.8795	1.010 (0.883–1.157)	0.8796	0.8795
CD4/CD8	1.204 (0.666–2.175)	0.5590	0.5384	1.204 (0.666–2.175)	0.5590	0.5384
log(IgE+1)	0.637 (0.405–1.002)	0.0559	0.0511	0.637 (0.405–1.002)	0.0559	0.0511
Age	0.538 (0.371–0.782)	<0.001	0.0011	0.538 (0.371–0.782)	<0.001	0.0011
Male sex	1.862 (0.585–5.929)	0.2888	0.2928	1.862 (0.585–5.929)	0.2888	0.2928

Table S4A. Complete primary multivariable results

All coefficients are reported, including non-significant estimates. Cell counts: OR per 100 cells/μL; percentages: per one percentage point; age: per year; male sex: versus female. CI type is labelled. PLR p-values govern the manuscript interpretation. These p-values are not multiplicity-adjusted.

Asthma

Predictor	n / events	OR (95% CI)	CI type	PLR p	Wald p	VIF
CD4+	201/92	0.979 (0.935–1.025)	Wald	0.3467	0.3581	2.210
CD19+	201/92	0.969 (0.918–1.022)	Wald	0.2283	0.2447	1.559
NK	201/92	1.008 (0.921–1.104)	Wald	0.8520	0.8548	1.400
CD4/CD8	201/92	0.811 (0.567–1.159)	Wald	0.2285	0.2504	1.461
log(IgE+1)	201/92	1.100 (0.903–1.340)	Wald	0.3316	0.3439	1.032
Age	201/92	0.993 (0.967–1.021)	Wald	0.6233	0.6391	1.100
Male sex	201/92	0.774 (0.432–1.386)	Wald	0.3803	0.3886	1.024

Allergic rhinitis

Predictor	n / events	OR (95% CI)	CI type	PLR p	Wald p	VIF
CD4+	201/117	0.923 (0.876–0.969)	Profile	<0.001	0.0019	2.210
CD19+	201/117	0.971 (0.919–1.026)	Wald	0.2826	0.2943	1.559
NK	201/117	1.032 (0.940–1.134)	Wald	0.4998	0.5061	1.400
CD4/CD8	201/117	1.077 (0.747–1.553)	Wald	0.6810	0.6909	1.461
log(IgE+1)	201/117	1.082 (0.880–1.331)	Wald	0.4464	0.4559	1.032
Age	201/117	0.995 (0.967–1.024)	Wald	0.7181	0.7271	1.100
Male sex	201/117	0.881 (0.476–1.629)	Wald	0.6811	0.6858	1.024

Atopic dermatitis

Predictor	n / events	OR (95% CI)	CI type	PLR p	Wald p	VIF
CD4+	201/56	1.044 (0.995–1.095)	Wald	0.0707	0.0825	2.210
CD19+	201/56	1.023 (0.965–1.083)	Wald	0.4407	0.4493	1.559
NK	201/56	1.014 (0.922–1.114)	Wald	0.7722	0.7782	1.400
CD4/CD8	201/56	0.938 (0.638–1.379)	Wald	0.7351	0.7448	1.461
log(IgE+1)	201/56	1.475 (1.167–1.904)	Profile	<0.001	0.0019	1.032
Age	201/56	0.956 (0.903–1.013)	Wald	0.0695	0.1250	1.100
Male sex	201/56	0.894 (0.457–1.749)	Wald	0.7423	0.7445	1.024

Food allergy

Predictor	n / events	OR (95% CI)	CI type	PLR p	Wald p	VIF
CD4+	201/57	1.034 (0.987–1.084)	Wald	0.1432	0.1595	2.210
CD19+	201/57	1.060 (1.004–1.120)	Profile	0.0341	0.0394	1.559

Predictor	n / events	OR (95% CI)	CI type	PLR p	Wald p	VIF
NK	201/57	0.938 (0.852–1.033)	Wald	0.1697	0.1919	1.400
CD4/CD8	201/57	1.138 (0.788–1.644)	Wald	0.4787	0.4904	1.461
log(IgE+1)	201/57	1.064 (0.854–1.325)	Wald	0.5712	0.5789	1.032
Age	201/57	0.983 (0.945–1.023)	Wald	0.3720	0.4106	1.100
Male sex	201/57	1.077 (0.557–2.085)	Wald	0.8236	0.8255	1.024

Wheezy-child phenotype

Predictor	n / events	OR (95% CI)	CI type	PLR p	Wald p	VIF
CD4+	201/16	0.991 (0.922–1.065)	Wald	0.8058	0.8060	2.210
CD19+	201/16	0.942 (0.861–1.030)	Wald	0.1766	0.1923	1.559
NK	201/16	1.010 (0.883–1.157)	Wald	0.8796	0.8795	1.400
CD4/CD8	201/16	1.204 (0.666–2.175)	Wald	0.5590	0.5384	1.461
log(IgE+1)	201/16	0.637 (0.405–1.002)	Wald	0.0559	0.0511	1.032
Age	201/16	0.538 (0.342–0.762)	Profile	<0.001	0.0011	1.100
Male sex	201/16	1.862 (0.585–5.929)	Wald	0.2888	0.2928	1.024

Table S4B. Complete univariate results

All coefficients are reported, including non-significant estimates. Cell counts: OR per 100 cells/µL; percentages: per one percentage point; age: per year; male sex: versus female. CI type is labelled. PLR p-values govern the manuscript interpretation. These p-values are not multiplicity-adjusted.

Asthma

Predictor	n / events	OR (95% CI)	CI type	PLR p	Wald p	VIF
CD3+	206/94	0.974 (0.951–0.996)	Profile	0.0220	0.0298	1.000
CD4+	206/94	0.958 (0.926–0.988)	Profile	0.0052	0.0092	1.000
CD8+	206/94	1.014 (0.965–1.065)	Wald	0.5790	0.5896	1.000
CD19+	206/94	0.957 (0.916–0.997)	Profile	0.0332	0.0410	1.000
NK	206/94	0.974 (0.906–1.047)	Wald	0.4597	0.4710	1.000
Total lymphocytes	206/94	0.985 (0.970–0.999)	Profile	0.0412	0.0496	1.000
CD4/CD8	206/94	0.725 (0.519–0.972)	Profile	0.0311	0.0442	1.000
log(IgE+1)	201/92	1.113 (0.921–1.344)	Wald	0.2622	0.2690	1.000
Age	206/94	1.004 (0.978–1.030)	Wald	0.7666	0.7737	1.000
Male sex	206/94	0.833 (0.478–1.450)	Wald	0.5157	0.5179	1.000

Allergic rhinitis

Predictor	n / events	OR (95% CI)	CI type	PLR p	Wald p	VIF
CD3+	206/120	0.946 (0.920–0.971)	Profile	<0.001	<0.001	1.000
CD4+	206/120	0.917 (0.882–0.950)	Profile	<0.001	<0.001	1.000
CD8+	206/120	0.931 (0.878–0.981)	Profile	0.0065	0.0110	1.000
CD19+	206/120	0.927 (0.885–0.966)	Profile	<0.001	<0.001	1.000
NK	206/120	0.932 (0.866–1.004)	Wald	0.0532	0.0628	1.000
Total lymphocytes	206/120	0.969 (0.953–0.984)	Profile	<0.001	<0.001	1.000
CD4/CD8	206/120	0.785 (0.589–1.047)	Wald	0.0850	0.0992	1.000
log(IgE+1)	201/117	1.099 (0.909–1.329)	Wald	0.3233	0.3291	1.000
Age	206/120	1.014 (0.986–1.043)	Wald	0.2897	0.3182	1.000
Male sex	206/120	0.975 (0.557–1.709)	Wald	0.9302	0.9306	1.000

Atopic dermatitis

Predictor	n / events	OR (95% CI)	CI type	PLR p	Wald p	VIF
CD3+	206/59	1.035 (1.011–1.061)	Profile	0.0034	0.0055	1.000
CD4+	206/59	1.053 (1.022–1.090)	Profile	<0.001	0.0016	1.000
CD8+	206/59	1.032 (0.979–1.087)	Wald	0.2324	0.2439	1.000
CD19+	206/59	1.056 (1.013–1.103)	Profile	0.0099	0.0115	1.000
NK	206/59	1.065 (0.987–1.149)	Wald	0.0964	0.1029	1.000
Total lymphocytes	206/59	1.024 (1.008–1.040)	Profile	0.0023	0.0034	1.000
CD4/CD8	206/59	1.185 (0.886–1.586)	Wald	0.2434	0.2524	1.000
log(IgE+1)	201/56	1.359 (1.093–1.713)	Profile	0.0053	0.0075	1.000
Age	206/59	0.946 (0.884–0.988)	Profile	0.0082	0.0379	1.000
Male sex	206/59	0.989 (0.537–1.820)	Wald	0.9715	0.9716	1.000

Urticaria

Predictor	n / events	OR (95% CI)	CI type	PLR p	Wald p	VIF
CD3+	206/11	1.002 (0.959–1.048)	Wald	0.9238	0.9189	1.000
CD4+	206/11	0.991 (0.930–1.055)	Wald	0.7890	0.7771	1.000
CD8+	206/11	0.960 (0.849–1.086)	Wald	0.5403	0.5169	1.000
CD19+	206/11	0.949 (0.855–1.054)	Wald	0.3120	0.3272	1.000
NK	206/11	0.943 (0.791–1.125)	Wald	0.5244	0.5137	1.000

Predictor	n / events	OR (95% CI)	CI type	PLR p	Wald p	VIF
Total lymphocytes	206/11	0.990 (0.957–1.023)	Wald	0.5369	0.5298	1.000
CD4/CD8	206/11	1.199 (0.728–1.974)	Wald	0.5067	0.4770	1.000
log(IgE+1)	201/11	1.469 (0.953–2.265)	Wald	0.0784	0.0816	1.000
Age	206/11	1.029 (0.990–1.069)	Wald	0.1753	0.1447	1.000
Male sex	206/11	0.859 (0.265–2.785)	Wald	0.7996	0.8001	1.000

Food allergy

Predictor	n / events	OR (95% CI)	CI type	PLR p	Wald p	VIF
CD3+	206/60	1.042 (1.018–1.070)	Profile	<0.001	0.0011	1.000
CD4+	206/60	1.057 (1.025–1.094)	Profile	<0.001	<0.001	1.000
CD8+	206/60	1.035 (0.983–1.091)	Wald	0.1806	0.1927	1.000
CD19+	206/60	1.076 (1.031–1.125)	Profile	<0.001	0.0010	1.000
NK	206/60	1.010 (0.935–1.090)	Wald	0.8055	0.8082	1.000
Total lymphocytes	206/60	1.024 (1.009–1.041)	Profile	0.0016	0.0026	1.000
CD4/CD8	206/60	1.390 (1.045–1.886)	Profile	0.0236	0.0298	1.000
log(IgE+1)	201/57	1.024 (0.832–1.259)	Wald	0.8237	0.8252	1.000
Age	206/60	0.959 (0.907–0.995)	Profile	0.0240	0.0621	1.000
Male sex	206/60	1.030 (0.561–1.891)	Wald	0.9244	0.9248	1.000

Wheezy-child phenotype

Predictor	n / events	OR (95% CI)	CI type	PLR p	Wald p	VIF
CD3+	206/16	1.027 (0.995–1.061)	Wald	0.1113	0.1004	1.000
CD4+	206/16	1.038 (0.997–1.081)	Wald	0.0801	0.0730	1.000
CD8+	206/16	1.010 (0.926–1.100)	Wald	0.8333	0.8259	1.000
CD19+	206/16	1.030 (0.965–1.099)	Wald	0.3959	0.3811	1.000
NK	206/16	1.036 (0.920–1.166)	Wald	0.5772	0.5629	1.000
Total lymphocytes	206/16	1.019 (0.997–1.041)	Wald	0.1090	0.0986	1.000
CD4/CD8	206/16	1.341 (0.899–2.001)	Wald	0.1685	0.1508	1.000
log(IgE+1)	201/16	0.635 (0.447–0.889)	Profile	0.0080	0.0089	1.000
Age	206/16	0.557 (0.380–0.751)	Profile	<0.001	<0.001	1.000
Male sex	206/16	1.590 (0.550–4.594)	Wald	0.3797	0.3918	1.000

Primary immunodeficiency

Predictor	n / events	OR (95% CI)	CI type	PLR p	Wald p	VIF
CD3+	206/7	1.003 (0.951–1.058)	Wald	0.9230	0.9135	1.000
CD4+	206/7	1.028 (0.971–1.088)	Wald	0.3990	0.3453	1.000
CD8+	206/7	0.965 (0.832–1.118)	Wald	0.6782	0.6330	1.000
CD19+	206/7	1.062 (0.977–1.153)	Wald	0.1893	0.1577	1.000
NK	206/7	1.043 (0.886–1.228)	Wald	0.6479	0.6127	1.000
Total lymphocytes	206/7	1.009 (0.976–1.042)	Wald	0.6395	0.6083	1.000
CD4/CD8	206/7	1.426 (0.856–2.376)	Wald	0.2172	0.1726	1.000
log(IgE+1)	201/7	0.823 (0.515–1.315)	Wald	0.4349	0.4148	1.000
Age	206/7	0.699 (0.488–1.000)	Wald	0.0519	0.0501	1.000
Male sex	206/7	3.302 (0.542–20.121)	Wald	0.1490	0.1951	1.000

Table S5A. Percentage sensitivity models

All coefficients are reported, including non-significant estimates. Cell counts: OR per 100 cells/µL; percentages: per one percentage point; age: per year; male sex: versus female. CI type is labelled. PLR p-values govern the manuscript interpretation. These p-values are not multiplicity-adjusted.

Asthma

Predictor	n / events	OR (95% CI)	CI type	PLR p	Wald p	VIF
CD4+ percentage	201/92	1.013 (0.986–1.041)	Wald	0.3325	0.3432	1.643
CD19+ percentage	201/92	0.990 (0.953–1.029)	Wald	0.6012	0.6115	1.130
NK percentage	201/92	1.004 (0.947–1.063)	Wald	0.8997	0.9021	1.098
CD4/CD8	201/92	0.660 (0.445–0.979)	Wald	0.0275	0.0388	1.673
log(IgE+1)	201/92	1.109 (0.912–1.348)	Wald	0.2886	0.3013	1.035
Age	201/92	0.999 (0.973–1.027)	Wald	0.9630	0.9646	1.056
Male sex	201/92	0.782 (0.438–1.396)	Wald	0.3962	0.4052	1.028

Allergic rhinitis

Predictor	n / events	OR (95% CI)	CI type	PLR p	Wald p	VIF
CD4+ percentage	201/117	0.992 (0.966–1.019)	Wald	0.5621	0.5732	1.643
CD19+ percentage	201/117	0.982 (0.945–1.021)	Wald	0.3495	0.3639	1.130
NK percentage	201/117	1.008 (0.951–1.069)	Wald	0.7736	0.7789	1.098
CD4/CD8	201/117	0.856 (0.595–1.233)	Wald	0.3878	0.4047	1.673

Predictor	n / events	OR (95% CI)	CI type	PLR p	Wald p	VIF
log(IgE+1)	201/117	1.089 (0.896–1.323)	Wald	0.3802	0.3924	1.035
Age	201/117	1.009 (0.981–1.037)	Wald	0.5164	0.5404	1.056
Male sex	201/117	0.909 (0.508–1.629)	Wald	0.7451	0.7495	1.028

Atopic dermatitis

Predictor	n / events	OR (95% CI)	CI type	PLR p	Wald p	VIF
CD4+ percentage	201/56	1.001 (0.972–1.031)	Wald	0.9364	0.9379	1.643
CD19+ percentage	201/56	1.007 (0.963–1.054)	Wald	0.7433	0.7453	1.130
NK percentage	201/56	1.004 (0.941–1.071)	Wald	0.9090	0.9102	1.098
CD4/CD8	201/56	1.126 (0.753–1.684)	Wald	0.5531	0.5627	1.673
log(IgE+1)	201/56	1.416 (1.118–1.795)	Wald	0.0024	0.0040	1.035
Age	201/56	0.935 (0.875–0.998)	Wald	0.0100	0.0424	1.056
Male sex	201/56	0.879 (0.457–1.691)	Wald	0.6962	0.6991	1.028

Food allergy

Predictor	n / events	OR (95% CI)	CI type	PLR p	Wald p	VIF
CD4+ percentage	201/57	0.999 (0.971–1.028)	Wald	0.9297	0.9313	1.643
CD19+ percentage	201/57	1.025 (0.982–1.069)	Wald	0.2520	0.2596	1.130
NK percentage	201/57	0.956 (0.895–1.022)	Wald	0.1745	0.1868	1.098
CD4/CD8	201/57	1.322 (0.896–1.950)	Wald	0.1468	0.1599	1.673
log(IgE+1)	201/57	1.050 (0.847–1.302)	Wald	0.6518	0.6571	1.035
Age	201/57	0.972 (0.932–1.014)	Wald	0.1306	0.1864	1.056
Male sex	201/57	1.029 (0.540–1.964)	Wald	0.9290	0.9297	1.028

Wheezy-child phenotype

Predictor	n / events	OR (95% CI)	CI type	PLR p	Wald p	VIF
CD4+ percentage	201/16	0.973 (0.927–1.022)	Wald	0.2570	0.2740	1.643
CD19+ percentage	201/16	0.923 (0.854–0.997)	Wald	0.0364	0.0416	1.130
NK percentage	201/16	0.984 (0.893–1.085)	Wald	0.7417	0.7463	1.098
CD4/CD8	201/16	1.381 (0.757–2.518)	Wald	0.3079	0.2922	1.673
log(IgE+1)	201/16	0.651 (0.420–1.011)	Wald	0.0564	0.0558	1.035
Age	201/16	0.571 (0.411–0.794)	Wald	<0.001	<0.001	1.056
Male sex	201/16	2.059 (0.633–6.704)	Wald	0.2237	0.2302	1.028

Table S5B. Comorbidity-adjusted sensitivity models

All coefficients are reported, including non-significant estimates. Cell counts: OR per 100 cells/µL; percentages: per one percentage point; age: per year; male sex: versus female. CI type is labelled. PLR p-values govern the manuscript interpretation. These p-values are not multiplicity-adjusted.

Asthma

Predictor	n / events	OR (95% CI)	CI type	PLR p	Wald p	VIF
CD4+	201/92	1.020 (0.961–1.083)	Wald	0.5177	0.5049	2.351
CD19+	201/92	0.977 (0.913–1.045)	Wald	0.4913	0.4941	1.610
NK	201/92	0.985 (0.882–1.100)	Wald	0.7876	0.7899	1.423
CD4/CD8	201/92	0.817 (0.530–1.259)	Wald	0.3368	0.3594	1.477
log(IgE+1)	201/92	1.207 (0.935–1.558)	Wald	0.1419	0.1481	1.161
Age	201/92	0.974 (0.941–1.008)	Wald	0.1122	0.1319	1.170
Male sex	201/92	0.724 (0.347–1.507)	Wald	0.3839	0.3877	1.036
Allergic rhinitis	201/92	2.085 (0.889–4.888)	Wald	0.0880	0.0909	1.635
Atopic dermatitis	201/92	0.200 (0.079–0.506)	Wald	<0.001	<0.001	1.459
Urticaria	201/92	0.063 (0.009–0.439)	Wald	<0.001	0.0052	1.106
Food allergy	201/92	0.184 (0.076–0.444)	Wald	<0.001	<0.001	1.388
Wheezy-child phenotype	201/92	0.019 (0.001–0.326)	Wald	<0.001	0.0064	1.290

Allergic rhinitis

Predictor	n / events	OR (95% CI)	CI type	PLR p	Wald p	VIF
CD4+	201/117	0.922 (0.866–0.981)	Wald	0.0081	0.0109	2.297
CD19+	201/117	0.986 (0.926–1.050)	Wald	0.6534	0.6639	1.611
NK	201/117	1.026 (0.916–1.149)	Wald	0.6505	0.6545	1.425
CD4/CD8	201/117	1.230 (0.791–1.914)	Wald	0.3399	0.3573	1.477
log(IgE+1)	201/117	1.195 (0.908–1.573)	Wald	0.1997	0.2046	1.165
Age	201/117	0.976 (0.945–1.008)	Wald	0.1297	0.1439	1.180
Male sex	201/117	0.923 (0.436–1.957)	Wald	0.8330	0.8354	1.038
Asthma	201/117	1.937 (0.813–4.611)	Wald	0.1286	0.1353	1.609
Atopic dermatitis	201/117	0.162 (0.065–0.406)	Wald	<0.001	<0.001	1.426
Urticaria	201/117	0.155 (0.032–0.739)	Wald	0.0129	0.0194	1.152
Food allergy	201/117	0.265 (0.109–0.645)	Wald	0.0027	0.0035	1.443

Predictor	n / events	OR (95% CI)	CI type	PLR p	Wald p	VIF
Wheezy-child phenotype	201/117	0.091 (0.019–0.428)	Wald	<0.001	0.0024	1.355

Atopic dermatitis

Predictor	n / events	OR (95% CI)	CI type	PLR p	Wald p	VIF
CD4+	201/56	1.019 (0.960–1.082)	Wald	0.5280	0.5325	2.356
CD19+	201/56	1.004 (0.936–1.077)	Wald	0.9127	0.9125	1.613
NK	201/56	1.026 (0.917–1.148)	Wald	0.6586	0.6600	1.425
CD4/CD8	201/56	0.999 (0.643–1.553)	Wald	0.9979	0.9980	1.486
log(IgE+1)	201/56	1.713 (1.241–2.364)	Wald	<0.001	0.0011	1.095
Age	201/56	0.961 (0.916–1.010)	Wald	0.0792	0.1151	1.161
Male sex	201/56	0.762 (0.339–1.712)	Wald	0.5103	0.5107	1.037
Asthma	201/56	0.218 (0.085–0.563)	Wald	0.0011	0.0016	1.533
Allergic rhinitis	201/56	0.198 (0.081–0.479)	Wald	<0.001	<0.001	1.522
Urticaria	201/56	0.069 (0.009–0.552)	Wald	0.0023	0.0118	1.122
Food allergy	201/56	1.176 (0.459–3.015)	Wald	0.7332	0.7359	1.517
Wheezy-child phenotype	201/56	0.142 (0.027–0.731)	Wald	0.0082	0.0196	1.363

Food allergy

Predictor	n / events	OR (95% CI)	CI type	PLR p	Wald p	VIF
CD4+	201/57	1.026 (0.965–1.090)	Wald	0.4104	0.4097	2.354
CD19+	201/57	1.042 (0.975–1.113)	Wald	0.2218	0.2260	1.595
NK	201/57	0.916 (0.819–1.025)	Wald	0.1079	0.1244	1.404
CD4/CD8	201/57	1.222 (0.789–1.893)	Wald	0.3710	0.3681	1.483
log(IgE+1)	201/57	1.006 (0.764–1.326)	Wald	0.9632	0.9635	1.175
Age	201/57	0.977 (0.940–1.014)	Wald	0.1824	0.2222	1.171
Male sex	201/57	1.193 (0.550–2.587)	Wald	0.6513	0.6545	1.038
Asthma	201/57	0.220 (0.091–0.530)	Wald	<0.001	<0.001	1.505
Allergic rhinitis	201/57	0.290 (0.118–0.713)	Wald	0.0059	0.0070	1.591
Atopic dermatitis	201/57	1.173 (0.452–3.045)	Wald	0.7385	0.7428	1.566
Urticaria	201/57	0.192 (0.033–1.124)	Wald	0.0435	0.0673	1.161
Wheezy-child phenotype	201/57	0.038 (0.005–0.288)	Wald	<0.001	0.0015	1.296

Wheezy-child phenotype

Predictor	n / events	OR (95% CI)	CI type	PLR p	Wald p	VIF
CD4+	201/16	1.048 (0.958–1.147)	Wald	0.3491	0.3068	2.353
CD19+	201/16	0.960 (0.860–1.071)	Wald	0.4891	0.4625	1.609
NK	201/16	0.989 (0.847–1.154)	Wald	0.8884	0.8848	1.423
CD4/CD8	201/16	0.859 (0.406–1.818)	Wald	0.7058	0.6907	1.486
log(IgE+1)	201/16	0.594 (0.308–1.147)	Wald	0.1697	0.1208	1.168
Age	201/16	0.691 (0.482–0.990)	Wald	0.0499	0.0441	1.126
Male sex	201/16	1.663 (0.401–6.898)	Wald	0.5144	0.4834	1.034
Asthma	201/16	0.028 (0.002–0.360)	Wald	<0.001	0.0061	1.463
Allergic rhinitis	201/16	0.464 (0.089–2.410)	Wald	0.3939	0.3609	1.561
Atopic dermatitis	201/16	0.257 (0.050–1.328)	Wald	0.1053	0.1049	1.472
Urticaria	201/16	0.073 (0.002–2.993)	Wald	0.0679	0.1674	1.141
Food allergy	201/16	0.066 (0.009–0.500)	Wald	0.0043	0.0085	1.355

Comorbidity models include the seven primary covariates plus the five other allergic/respiratory labels. Primary immunodeficiency is not a comorbidity covariate. Sparse outcomes, including 16 wheezy-child cases, make these expanded models exploratory; low VIF does not remove overfitting risk.

Table S6. Exploratory threshold classification counts

Phenotype / marker	Positive threshold	TP	FN	FP	TN	Sensitivity %	Specificity %
Asthma / CD4+	<2787.6	83	11	78	34	88.3	30.4
Allergic rhinitis / CD4+	<2353.8	93	27	37	49	77.5	57.0
Atopic dermatitis / CD4+	>2539.8	31	28	32	115	52.5	78.2
Urticaria / CD19+	<859.6	6	5	56	139	54.5	71.3
Food allergy / CD4+	>2787.6	23	37	22	124	38.3	84.9
Wheezy-child phenotype / Total lymphocytes	>6371.0	9	7	45	145	56.2	76.3
Primary immunodeficiency / CD19+	>1292.1	6	1	78	121	85.7	60.8

TP/FN/FP/TN refer to the recorded clinical label at the data-selected threshold. They are not independent validation results. The underlying threshold values, before rounding for display, were used for classification. CIs and AUCs are in main Table 6.
