# Supplementary material for: Epigenetic Lymphocyte Subset Associations Across Allergic Phenotypes in a Predominantly Pediatric Cohort
Source: Diagnostics (Basel). 2026 Oct 21;16(20):3432. doi: 10.3390/diagnostics16203432 (PMC13703722; doi:10.3390/diagnostics16203432)
Supplement: Supplementary file 1 [file diagnostics-16-03432-s001.zip › File S2 STARD Checklist.pdf]

STARD 2015 Checklist

Manuscript: "Distinct epigenetic lymphocyte subset signatures across pediatric allergic phenotypes." The "Page/§" column refers to sections of the submitted manuscript; please update page numbers to match the typeset PDF before final submission.

No.	Item (STARD 2015)	Page/§
**TITLE OR ABSTRACT**		
1	Identification as a study of diagnostic accuracy using at least one measure of accuracy (e.g., sensitivity, specificity, predictive values, or AUC)	Title; Abstract
**ABSTRACT**		
2	Structured summary of study design, methods, results, and conclusions	p.1 (Abstract)
**INTRODUCTION**		
3	Scientific and clinical background, including the intended use and clinical role of the index test	Introduction
4	Study objectives and hypotheses	Introduction (final ¶)
**METHODS**		
5	Whether data collection was planned before the index test and reference standard were performed (prospective) or after (retrospective)	§2.1 (retrospective)
6	Eligibility criteria	§2.1
7	On what basis potentially eligible participants were identified (e.g., symptoms, prior test results, registry)	§2.1
8	Where and when potentially eligible participants were identified (setting, location, dates)	§2.1
9	Whether participants formed a consecutive, random, or convenience series	§2.1
10a	Index test, in sufficient detail to allow replication	§2.3 (i.Mune TBNK)
10b	Reference standard, in sufficient detail to allow replication	§2.2 (EAACI-based clinical diagnosis)
11	Rationale for choosing the reference standard	§2.2
12a	Definition of and rationale for test positivity cut-offs or result categories of the index test, distinguishing pre-specified from exploratory	§2.6 (Youden J; exploratory)
12b	Definition of and rationale for test positivity cut-offs or result categories of the reference standard, distinguishing pre-specified from exploratory	§2.2 (binary phenotypes)
13a	Whether clinical information and reference standard results were available to the performers/readers of the index test	§2.1/§2.3 (record-based)
13b	Whether clinical information and index test results were available to the assessors of the reference standard	§2.2
14	Methods for estimating or comparing measures of diagnostic accuracy	§2.6
15	How indeterminate index test or reference standard results were handled	§2.1 (exclusions)
16	How missing data on the index test and reference standard were handled	§2.1 (excluded)
17	Any analyses of variability in diagnostic accuracy, distinguishing pre-specified from exploratory	§2.6/§3 (age; exploratory)
18	Intended sample size and how it was determined	N/A — convenience sample (see Limitations)
**RESULTS**		
19	Flow of participants, using a diagram	§3.1 (n = 206; text)
20	Baseline demographic and clinical characteristics of participants	§3.1; Table 1
21a	Distribution of severity of disease in those with the target condition	§3.1; Tables 1–2
21b	Distribution of alternative diagnoses in those without the target condition	Table 1B
22	Time interval and any clinical interventions between index test and reference standard	§2.1 (contemporaneous records)
23	Cross tabulation of the index test results by the results of the reference standard	Tables 2–4
24	Estimates of diagnostic accuracy and their precision (e.g., 95% confidence intervals)	Table 4 (AUC with 95% CI)

25	Any adverse events from performing the index test or the reference standard	None (non-interventional)
**DISCUSSION**		
26	Study limitations, including sources of potential bias, statistical uncertainty, and generalisability	§4 (Limitations ¶)
27	Implications for practice, including the intended use and clinical role of the index test	§4; §5
**OTHER INFORMATION**		
28	Registration number and name of registry	N/A — retrospective, non-interventional
29	Where the full study protocol can be accessed	From corresponding author
30	Sources of funding and other support; role of funders	Funding (none)
